# Understanding societal challenges: a Neurotech^EU^ perspective

**DOI:** 10.3389/fnins.2024.1330470

**Published:** 2024-07-26

**Authors:** Daniela Schulz, Carmen Lillo-Navarro, Marc Slors, Anett Hrabéczy, Martin Reuter

**Affiliations:** ^1^Behavioral Biology Laboratory, Institute of Biomedical Engineering and Center for Life Sciences and Technologies, Boğaziçi University, Istanbul, Türkiye; ^2^Department of Pathology and Surgery, Center for Translational Research in Physiotherapy, Miguel Hernández University, Alicante, Spain; ^3^Philosophy of Mind and Cognition, Faculty of Philosophy, Theology and Religious Studies, Radboud University, Nijmegen, Netherlands; ^4^Department of Educational Studies, Institute of Educational Studies and Cultural Management, University of Debrecen, Debrecen, Hungary; ^5^Personality Psychology and Biological Psychology, Laboratory of Neurogenetics, Department of Psychology, University of Bonn, Bonn, Germany

**Keywords:** European University, neuro-technologies, societal innovation, public opinion, policy-making, ethics, personality traits, survey

## Abstract

Futuristic universities like The Neurotech^EU^ and the technological innovations they provide will shape and serve society, but will also require support from society. Positive attitudes about neuro-technologies will increase their reach within society and may also impact policy-making, including funding decisions. However, the acceptability rates, especially of invasive neuro-technologies, are quite low and the majority of people are more worried than enthusiastic about them. The question therefore arises as to what neuro-technological advances should entail. In a rare effort to reach out to the public, we propose to conduct a trans-national survey with the goal to better understand the challenges of our Neurotech^EU^ nations. We aim to compare and contrast our nations specifically with respect to their perspectives on neuro-technological advances, i.e., their needs for, interests in, access to, knowledge of and trust in neuro-technologies, and whether these should be regulated. To this end, we have developed the first version of a new tool—the *Understanding Societal Challenges Questionnaire (USCQ)—*which assesses all six of these dimensions (needs, interest, access, knowledge, trust, and policy-making) and is designed for administration across EU/AC countries. In addition to trans-national comparisons, we will also examine the links of our nations' perspectives on neuro-technological advances to demographic and personality variables, for example, education and socio-economic status, size of the residential area, the Big Five personality traits, religiosity, political standings, and more. We expect that this research will provide a deeper understanding of the challenges that our nations are facing as well as the similarities and differences between them, and will also help uncover the variables that predict positive and negative attitudes toward neuro-technological advances. By integrating this knowledge into the scientific process, The Neurotech^EU^ may be able to develop neuro-technologies that people really care about, are ethical and regulated, and actually understood by the user.

## 1 Introduction

Neuro-technologies are tools and methods as diverse as cochlear implants, neuroimaging, deep brain stimulation, drug delivery systems and pharmaceuticals, machine learning and artificial intelligence, digital medicine and wearable sensors, mobile apps, and virtual reality games (Sveistrup, 2004; Friston, 2009; Macherey and Carlyon, 2014; Elenko et al., 2015; Habets et al., 2018, 2020; Heijmans et al., 2019; Adepu and Ramakrishna, 2021; Berisha et al., 2021; Chen et al., 2022; Park et al., 2022; Abd-alrazaq et al., 2023). Neuro-technologies are employed to diagnose and treat medical conditions like Parkinson's disease, stroke, chronic pain, obesity and depression, but can also help to prevent disease or enhance life quality by improving sleep and attention, relieving stress, supporting weight loss, and reducing the risk of falls in the elderly (Anguera et al., 2013; Cheatham et al., 2017; Habets et al., 2018; Chen et al., 2019, 2022; Tegeler et al., 2020; Abd-alrazaq et al., 2023; Fisher and Lempka, 2023; Wang et al., 2023). Neuro-technologies are also predicted to have economic growth potential (Garden et al., 2019; Neurotech Reports, 2022), attesting to their significance for society.

**Figure 1:**
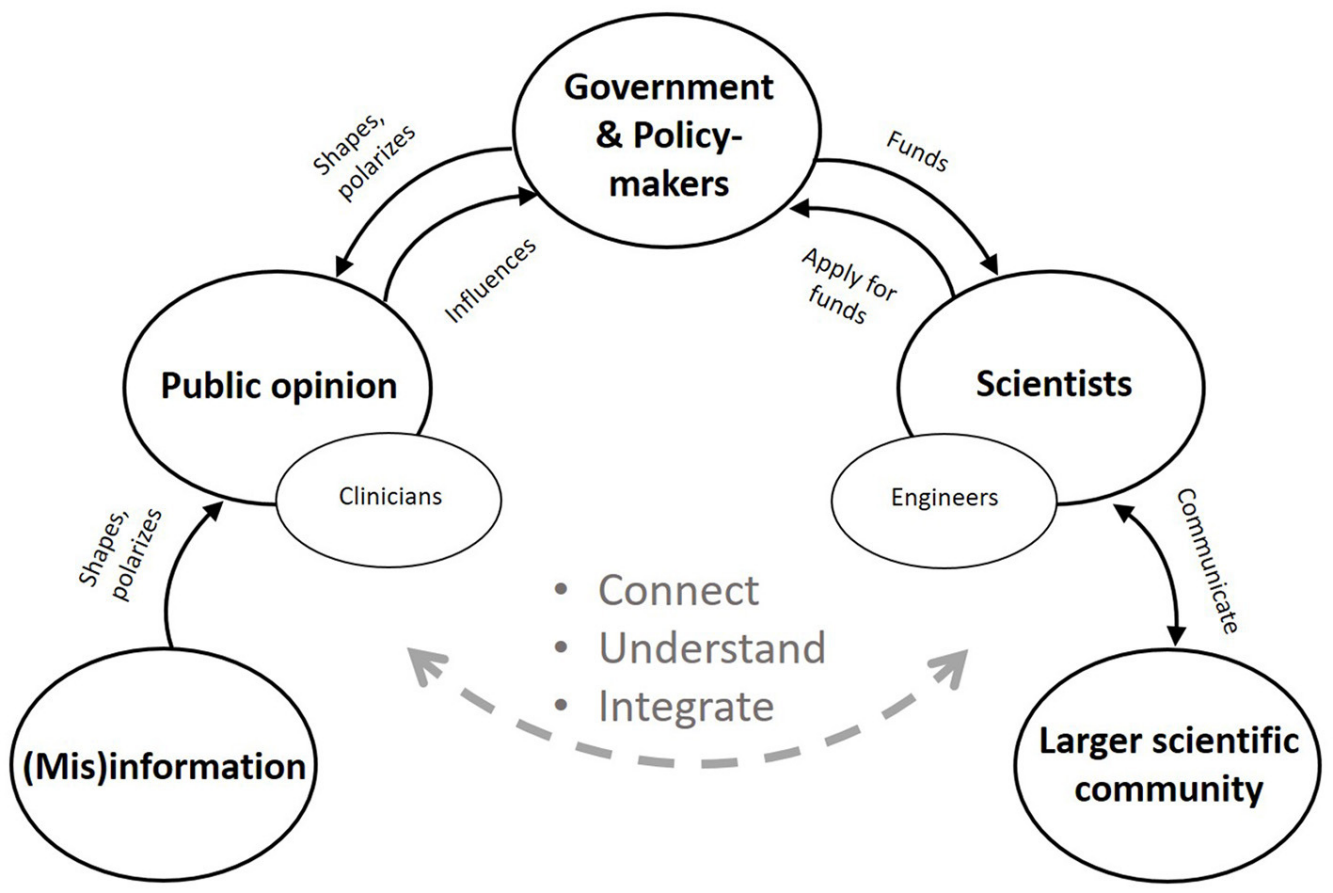
The scientist's web. Neuro-engineers and other innovators typically interact with government agencies to secure funding for research. They submit research proposals in response to specific calls that are based on programmatic priorities. Scientists also interact with each other to exchange ideas, for example, at scientific venues. Communication with the public is rare, however. Even engineers who develop the technologies and clinicians who apply them do not communicate enough (Weber, 2019). In the meantime, public opinion is shaped through public media, including misinformation spread online.

The European University of Brain and Technology (Neurotech^EU^) was funded by the European Commission to foster neuroscience education, research and innovation, and to generate societal impact through the development of new and improved neuro-technologies (https://theneurotech.eu/). Futuristic universities like Neurotech^EU^ and the technological innovations they provide will shape and serve society, but will also require support from society. That's because public opinion matters, trust in and thus acceptance of new technologies will determine consumer reach. Public opinion also influences policy-making, where salient topics with coherent opinions about them are more likely to become integrated into programmatic priorities (Burstein, 2003; Christian, 2008; Spendzharova and Versluis, 2013; Bromley-Trujillo and Karch, 2021). However, there seems to be a gap between science and the public (McFadden, 2016; Coates McCall et al., 2019). While neuroscientists, neuro-engineers and other innovators interact with government agencies to secure funding for research, and exchange ideas with each other, they typically do not reach out to the public to decide on the technologies they wish to develop (Figure 1). Even engineers and clinicians, who develop and apply the technologies respectively, do not communicate enough (Weber, 2019). In the meantime, public opinion is shaped through the media. Policy-makers themselves shape public opinion, but many other influences exist, including misinformation (fake news) spread online (Funk, 2020; Cacciatore, 2021). Therefore, it is important that scientists, too, connect with the public, understand their challenges, and integrate this knowledge into the scientific process.

Evidence suggests that the level at which the general public and patients, in particular, accept and welcome new neuro-technologies is variable. Sattler and Pietralla (2022) found, for example, that the moral acceptability rate and willingness to use brain stimulation devices were 25.5% and 28.7%, respectively, indicating that the majority of the participants—a representative sample of the adult German population—is not fully embracing this technology. The results were similar for brain-computer interfaces, the second type of technology examined. The use of these technologies for treatment was deemed more acceptable than their use for self-enhancement, and noninvasive applications were preferred over invasive ones. Sociodemographic characteristics, specifically, being female, older, and religious also contributed to a lower acceptance rate and/or willingness to use one or both technologies (Sattler and Pietralla, 2022). A US-based survey found that the public was much more worried than enthusiastic about gene editing, brain chips, and synthetic blood used for self-enhancement (Funk et al., 2016). While the interest in using assistive technologies was high in patients with spinal cord injuries, the acceptability rate of invasive technologies was still < 50% (Huggins et al., 2015). Non-invasive technologies are clearly preferred, but even these have their barriers in actually getting used; patients with Parkinson disease reported a low usability, discomfort or pain, and a lack of familiarity with such technologies (Laar et al., 2023).

As Neurotech^EU^, we will be confronted with many different attitudes about what neuro-technological advances should entail. Somewhat representative of the complexity that makes up the European Union (EU) and its Associated Member States (AC), our Neurotech^EU^countries—The Netherlands (NL), Spain (ES), Sweden (SE), Germany (DE), Türkiye (TR), Romania (RO), Hungary (HU), France (FR), and Iceland (IS) who are represented by Radboud University, Miguel Hernández University of Elche, Karolinska Institutet, University of Bonn, Boǧaziçi University, Iuliu Hatieganu University of Medicine and Pharmacy, University of Debrecen, University of Lille, and Reykjavik University, respectively—differ in social, cultural and individual characteristics that may translate into differences in opinion, both at the expert level and our broader societies. To serve everyone in the best ways possible, we therefore propose to conduct a trans-national study with the goal to better understand the challenges of our nations. We aim to compare and contrast our nations specifically with respect to their perspectives on neuro-technological advances, that is, their needs for, interests in, access to, knowledge of and trust in neuro-technologies, and whether these should be regulated. To our knowledge, no other trans-national studies have examined these variables before. We expect that, in the short-term, our study will provide a deeper understanding of the challenges that our nations are facing, the similarities and differences between our countries, and that through the process of science we will integrate our countries more. In the long run, we hope that our insights will benefit The Neurotech^EU^ in its efforts to develop neuro-technologies that people really care about, are accessible, useful, trusted, ethical, regulated, safe, research-based, new and proven, and are actually understood by the user. Connecting with the public, understanding their challenges, and integrating this knowledge into the scientific process may also result in a greater sense of inclusion and more excitement about the opportunities that come with research and innovation.

To achieve our goal, we have developed the first version of a new tool—the *Understanding Societal Challenges Questionnaire (USCQ)* which assesses people's perspectives on neuro-technologies by focusing on six question domains (needs, interest, access, knowledge, trust, and policy-making)—that can be administered across EU/AC nations. The USCQ has a fixed format that uses Likert scales for most items. It asks the respondents to rate their perspectives on neuro-technological advances more broadly, unlike other questionnaires which focus on a few specific technologies (e.g., Funk et al., 2016; Sattler and Pietralla, 2022). Because neuro-technologies are very diverse, and we are interested in measuring general attitudes of acceptability, it is our intention to not bias the respondents toward a specific topic. We hypothesize that the structure of the USCQ is formed by six latent variables that correlate predominantly with the respective items in the six question domains, irrespective of the nation that is measured. On the other hand, we expect that the domain means will vary across countries based on differing sociodemographic characteristics. For example, older age groups often feel barriers to the use of new technologies and are less accepting of these (Tacken et al., 2005; Sattler and Pietralla, 2022), and among our Neurotech^EU^ countries DE is the oldest with a median age of 44.91, whereas TR is the youngest with a median age of 31.76 (Database.earth, 2024). By this criterion, TR is expected to have higher acceptability rates than DE. The link between religiosity and societal perspectives on neuro-technological advances is also expected to impact our research, as it was shown that people who identify as religious are less accepting of new neuro-technologies (Funk et al., 2016; Sattler and Pietralla, 2022). According to the (Inglehart-Welzel World Cultural Map - World Values Survey 7, 2023), TR and RO have relatively high scores on the traditional and survival dimensions which emphasize the importance of religion and economic and physical security, respectively, whereas countries like DE, NL and IS fall at the opposite side of the spectrum with high scores on the secular-rational and self-expression dimensions, and HU, ES and FR falling somewhere in between. On the other hand, countries that score high on the survival dimension like TR and RO report relatively poor health and high levels of faith in science and technology, which might make neuro-technological advances more acceptable. In recent years, the level of religiosity has declined in all but a handful of countries studied in the World Values Survey (Inglehart, 2020). However, people that do not identify as religious, may nevertheless identify as spiritual. Religiosity in Caucasian countries that are predominantly Christian was not predictive of the level of spirituality involving exemplary humanity and moral values (Worthington et al., 2003). Given this complexity, we wish to ask our Neurotech^EU^ countries about both religiosity and spirituality.

To identify the sociodemographic variables that best predict positive and negative attitudes about neuro-technological advancements, we developed the 20-item *Understanding Societal Characteristics Form (USCF)* which asks about age, gender, education, size of the residential area, religiosity, political standings, and more. We include a question on maternal education as it was shown that less schooling in mothers is a predictor of emotional symptoms, peer-and conduct-related problems, and hyperactivity-inattention in children and adolescent offspring, and of life stress and symptoms of depression and anxiety in young adults (Meyrosea et al., 2018; Swartz et al., 2018). On the other hand, a causal protective effect of maternal schooling on the mental health of children in late adolescence and adulthood was not found (Graeber and Schnitzlein, 2019). Instead, an extra year of middle school, caused by a schooling law reform in Germany, had a negative effect on the mental health of daughters, possibly due to a greater absence of the mothers from home, although it led to an increase in household resources. However, the negative impacts of this trade-off between reduced parenting time and higher earnings may not translate to non-Western countries like TR that value intergenerational ties and interdependence more than individualism, even in times of urbanization and increased financial autonomy (Kagitcibasi and Ataca, 2005; Kagitcibasi et al., 2010). Therefore, we predict an effect of maternal education on indicators of brain health and thus the acceptability of neuro-technological advances, albeit differentially for nations that are more individualist as opposed to interdependent.

Personality is also an important predictor of attitudes about health-related issues, such as mandatory vaccinations during the Coronavirus disease 2019 (COVID-19) pandemic and related restrictions imposed by the government (Lippold et al., 2020). Personality also predisposes to risky behavior (Reuter et al., 2002), which might influence perceptions on the use of risky technologies. Moreover, personality mediates the influence of political ideology on societal attitudes (Grünhage and Reuter, 2020), and predicts religiosity and a general disposition to trust (Ezirim et al., 2021). Trust has been defined as a belief about an individual's or group's trustworthiness under conditions of unknown outcomes (Robbins, 2016), and is considered absolutely essential for any aspect of societal functioning, including educational attainment, political participation, socio-economic development, and physical and psychological wellbeing (Crepaz and Polk, 2015; Hamamura et al., 2017; Garden et al., 2019; Ortiz-Ospina and Roser, 2020). It is thus conceivable that personality mediates or moderates, at least in part, several characteristics that predict societal attitudes about neuro-technological advances.

Taken together, our survey data will be used to compare and contrast our Neurotech^EU^ nations with regard to their attitudes about neuro-technological advances, and to determine the characteristics that predict positive and negative attitudes, respectively. While we have laid out a few specific hypotheses above, the study is considered mainly exploratory given the number of variables that will be examined, and is intended to provide a foundation for further explorations long-term, including the effects of interventions derived from knowledge of the present study.

## 2 Methods and analysis

### 2.1 Study design

**Figure 2:**
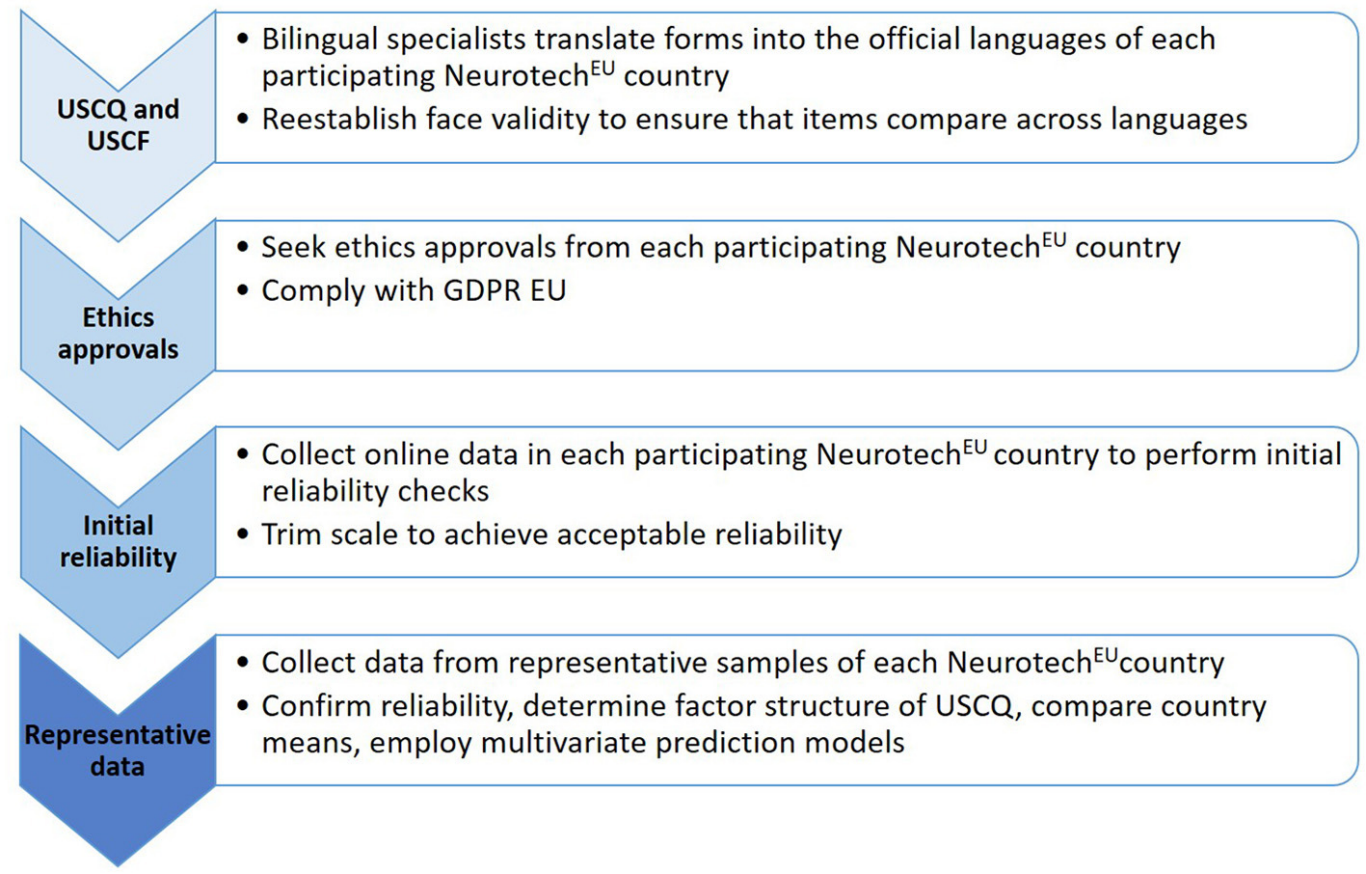
Study design. The progression of steps to be implemented next in Phase 1 of project “Understanding Societal Challenges: A Neurotech^EU^ perspective”. Phase 2 is planned as a follow-up study to assess longitudinal changes based on (e.g., educational) interventions that address the challenges of our societies. GDPR EU, General Data Protection Regulation EU (2016).

The design of our study is summarized in Figure 2. While we have established face validity of our forms in English language, these will be translated into the official languages of each participating Neurotech^EU^ country. Bilingual specialists will use back-and-forth translation to ensure that the translations are accurate and the meanings of the items comparable across languages. Next, ethics approvals will be sought by each participating Neurotech^EU^ country (refer to Section 4. Ethics and Dissemination). The translated forms will then be administered online for initial reliability testing of conceptually similar items. The English forms will be provided as a choice to the participants and tested alongside the translated versions in each country. The scales will be trimmed, if necessary, to achieve acceptable reliabilities (Cronbach's alpha > 0.70). Finally, we will collect field data from a representative sample of each Neurotech^EU^ country. Among other, the reliability analyses will be repeated, the factor structures of the USCQ determined, country means compared, and the influence of population characteristics on attitudes regarding neuro-technological advances assessed (refer to Section 2.6 Statistical Analyses). The results will be disseminated to the Neurotech^EU^ community and beyond.

### 2.2 Participants

Our participants will be 18 years of age or older. Females and males will be represented in about equal numbers. All education levels will be included for analysis (refer to Section 2.3 Understanding Societal Characteristics Form (USCF) for socio-demographic information). For initial reliability testing of our forms, we will recruit 100 participants per nation who will provide their responses online. These data will also be utilized to calculate the sample size of our final data set, which will consist of representative samples from each Neurotech^EU^ country. Based on other nation-wide surveys, we predict that ~1,000 data per nation will suffice to achieve representation (3M State of Science Index, 2022). The participants will be found in their day-to-day environments, both online and in the field, as appropriate. National statistics will be used to determine the proportion of internet users and non-users, age distributions and other socio-demographic characteristics of each sample. We will further classify our respondents into different stakeholder categories, such as patients, caregivers, clinicians, and company representatives, who would most directly benefit from neuro-technological advances (refer to USCF, item #7). Participants who cannot understand the questions due to cognitive impairments, will be excluded from analysis. Caregivers (and experimenters) can read the questions and record the answers for another person, if deemed necessary, for example due to sensory-motor impairments. If possible, random sampling procedures will be used for recruitment. Professional companies experienced in social research will be hired to support the data collection phase. The participants will not be promised any rewards a priori but will be offered a personality analysis as a free gift after the responses are received. The participants will provide written informed consent before data collection. All procedures will be approved by our local ethics committees and conducted in accordance with the Declaration of Helsinki and the directives of the General Data Protection Regulation EU (2016).

### 2.3 Understanding societal characteristics form (USCF)

To generate statistical data related to the population characteristics of our Neurotech^EU^ nations, we developed the 20-item-long USCF (Supplementary material 1), which asks about demographic variables and more, such as education and socio-economic status, size of the residential area, religiosity, and political standings. The USCF was designed for administration across EU/AC countries and is therefore taking the diverse educational systems, cultural norms, and sensitivities of our countries into account. We will use the information provided by this form to better understand the similarities and differences between our nations and to determine the characteristics that best predict positive and negative attitudes about neuro-technological advances.

### 2.4 Big Five personality traits

The “Big-Five” is the most accepted model of personality; it assumes that personality has five dimensions, i.e. openness to experience, conscientiousness, extraversion, agreeableness and neuroticism (Costa and McCrae, 1992). These dimensions can be assessed efficiently with the 10-item short version of the Big Five Inventory (BFI; Rammstedt and John, 2007), which we will employ in the present study. The factor structure of the BFI has been demonstrated to be invariant across countries and cultures, and its psychometric properties were found to be excellent (Kajonius and Mac Giolla, 2017). Personality is considered a stable trait. Accordingly, twin studies provided substantial heritability estimates of 0.40–0.60 for the Big-Five dimensions (Bouchard and McGue, 2003).

### 2.5 Understanding societal challenges questionnaire (USCQ)

The first version of the USCQ (Supplementary material 2) specifically asks about the respondent's perspectives on neuro-technological advances. The form has 30 items which cover 6 question domains—needs, interest, access, knowledge, trust, and policy-making. These domains contain 7, 4, 6, 3, 7, and 3 items, respectively. A Likert scale with five response categories is used as a format for most of the items, where 1 = strongly disagree, 2 = disagree, 3 = neither agree nor disagree, 4 = agree, and 5 = strongly agree, or where 1 = never, 2 = once a year, and 3 = once a month, 4 = once a week, and 5 = daily. Items 5, 6, and 14 have a dichotomous response format (yes/no). Item 5, for example, inquires whether the participant has any neurological condition. Four items (7, 16, 18, 19) use a multiple-select format. E.g. item 18 asks ‘Which of the following technologies are you familiar with?' and possible responses include PC, wearable technologies, machine learning, CRISPR-Cas9, and many others. Finally, item 20 (What do you feel about ‘neuro-technologies'? And to what extent?) uses a multiple-choice format in which the participants rate several different feelings on a 5-point Likert scale.

### 2.6 Statistical analyses

The reliability of the USCQ will be measured with Cronbach's alpha. The Likert-scale items in each domain—needs, interest, access, knowledge, trust, and policy-making—will be analyzed separately. Items with a low reliability (item-total correlation <0.25 and if item deleted, alpha increases) will be removed from the questionnaire and, if necessary, replaced by new items. Cronbach's alphas of >0.70 will be considered acceptable.

Once we obtained the field data from our representative samples, the factor structure of the USCQ will be determined. We will employ confirmatory factor analysis (CFA), in a structural equation model framework, to test the hypothesis that the structure of the USCQ is formed by six latent variables that correlate predominantly with the respective items in the six question domains of the USCQ. The fit between our theoretical model and the empirical data will be tested using fit indexes, including the chi-square test, root mean square error approximation (RMSEA), and the comparative fit index (CFI). We will employ second order CFA to determine a common higher-level factor, termed “General Acceptability of Neuro-Technological Advances” factor. Once this factor is clearly defined, and the items contributing to this general factor are known, we will compute a composite score for each participant, which will equal the sum of scores from each contributing item. The construct validity of the composite score will be tested in our ongoing researches. The CFAs will be applied to the data from each Neurotech^EU^ country. If found invariant, the data from all countries will be pooled for a final analysis.

All data measured on a ratio scale will be checked for normality and equal variance of the distributions, using the Kolmogorov-Smirnov test and Levene-statistic, respectively. If results allow for parametric testing to be used, group comparisons will be performed using ANOVA models. If the assumptions of normality and equal variance are violated, we will apply non-parametric statistics to the data. The Kruskal-Wallis test will be used to compare three or more groups. The Mann-Whitney U test will be used for *post-hoc* comparisons. Z-score statistics will be employed for standardization of the data across countries.

While we assume face validity of our measures at this point, we will probe into prediction and construct validity through simple correlation and multi-factorial analyses, including factor and structural equation analyses. To test for linear associations between the variables, we will apply the Pearson or Spearman rank-order correlation. Multivariate prediction models, i.e., path analysis and multiple regression will be used for mediation and moderation analyses. Continuous input variables will be centered to reduce multi-collinearity. These analyses will be conducted to identify the characteristics, e.g., demographic and personality variables, that reliably predict positive and negative attitudes about neuro-technologies.

All tests will be two-tailed and *P* ≤ 0.05 will be considered a measure of effect.

## 3 Discussion

In summary, this study was designed with the goal to better understand the challenges of our Neurotech^EU^ nations, specifically, their needs for, interests in, access to, knowledge of and trust in neuro-technologies, and whether these should be regulated. The data collected in each participating country will be used to determine the similarities and differences between our nations, and the characteristics that best predict positive and negative attitudes about neuro-technological advances. In the long run, the insights gained will benefit The Neurotech^EU^ in its efforts to develop neuro-technologies that people really care about, are accessible and understood by the user, are ethical, regulated and safe, based on research, and are new and clinically proven.

Studies have shown that healthy people as well as patients prefer non-invasive over invasive neuro-technologies (Huggins et al., 2015; Sattler and Pietralla, 2022). Surprisingly however, it is common that neuroscientists and neuro-engineers develop cutting-edge technologies that are highly invasive, but are considered the next frontier, and then face a myriad of challenges in translation (Weber, 2019; Shen et al., 2020). Also, despite their great potential, neuro-technologies used in preventive medicine have received much less attention than technologies that treat symptomatology (Elenko et al., 2015). Neuro-technologies that focus, for example, on sleep, diet, exercise and cognitive biases, which are often impacted early in the development of psychiatric and neurological diseases, might help prevent the transition from these early changes into full blown conditions that are hard-to-treat by the time clinical diagnoses are made (Schulz, 2020). Therefore, the NeurotechEU has a great opportunity to set its mark as a leader in the advancement of neuro-technologies that take attitudes of people into account, and focus on prevention and health in addition to (or more than) disease and treatment.

The proposed study is novel in several ways. Firstly, we are not aware of other trans-national studies that have asked the general population about their perspectives on neuro-technological advances across the six domains covered in the USCQ (needs, interest, access, knowledge, trust, and policy-making). Furthermore, we will focus our inquiry on a more general set of neuro-technologies rather than a few specific ones, as is common in other surveys (Funk et al., 2016; Sattler and Pietralla, 2022), because neuro-technologies are diverse, and we wish to be encompassing and avoid biases toward a specific topic. Previous studies have identified a few characteristics that predict positive and negative attitudes about health-related issues, including age, gender, religiosity, political standings, and personality (Funk et al., 2016; Grünhage and Reuter, 2020; Lippold et al., 2020; Sattler and Pietralla, 2022). Here, we will inquire about these variables and more, using the USCF which we devised for application across EU/AC countries, taking the diverse educational systems, cultural norms, and sensitivities of our countries into account. Compared to other cross-country studies, which collected their survey data online (e.g., Kajonius and Mac Giolla, 2017; Lippold et al., 2020), we aim to collect field data as necessary to achieve representation of each Neurotech^EU^ nation. National statistics will be used to determine the proportion of internet users and non-users, age distributions and other socio-demographic characteristics of each sample. While this is difficult to achieve, we will seek support from research companies specialized in collecting such data. Finally, to avoid sampling biases by including only participants who are fluent in English, we will translate our forms into the official languages of each participating Neurotech^EU^ country.

In conclusion, our attempt at bridging the gap between science and the public may result in neuro-technological advances that our broader societies will value more. We further expect to highlight the importance of non-invasive over invasive neuro-technologies, and technologies used in preventive medicine over those used to treat symptomatology. In the long run, the insights gained in the present study may benefit The Neurotech^EU^ in devising interventional (e.g. educational) strategies that aim to innovate our societies.

## Data availability statement

The original contributions presented in the study are included in the article/Supplementary material, further inquiries can be directed to the corresponding author.

## Ethics statement

This study has been designed to ensure compliance with the ethical principles set out by each participating nation as well as the international standards described in the Declaration of Helsinki (2013). It furthermore takes into account the directives provided by the General Data Protection Regulation EU (2016). Data collection in each participating Neurotech^EU^ country will commence only after all regulatory requirements and legal obligations have been assessed and ethics approvals were sought.

Before enrollment, all participants will be fully informed about the study. Only participants older than age 18 will be recruited. All participants will provide written informed consent to the processing of personal data in anonymous and aggregate form, by authorized personnel involved in the research, for up to 20 years from the conclusion of the study.

All data will be digitized, made anonymous, and archived on a centralized and secure IT platform in Germany or another EU member state involved in the study. The data will be coded with numbers and will not be associated with the name of the participant or any personally identifiable information.

The study findings will be used for publication in peer-reviewed scientific journals and presentations at scientific meetings. The data will also benefit The Neurotech^EU^ and its stakeholders in its mission to foster neuroscience education, research and innovation, and to generate societal impact through the development of neuro-technologies that are aligned with its values.

## Author contributions

DS: Conceptualization, Methodology, Project administration, Visualization, Writing—original draft, Writing—review & editing. CL-N: Conceptualization, Methodology, Writing—original draft. MS: Conceptualization, Methodology, Visualization, Writing—original draft. AH: Conceptualization, Methodology, Writing—original draft. MR: Conceptualization, Methodology, Visualization, Writing—original draft, Writing—review & editing.
