# Supplementary material for: Understanding societal challenges: a NeurotechEU perspective
Source: Front Neurosci. 2024 Jul 26;18:1330470. doi: 10.3389/fnins.2024.1330470 (PMC11313264; doi:10.3389/fnins.2024.1330470)
Supplement: Supplementary file 1 [file Table_1.DOCX]

Supplementary Material

Understanding societal challenges: a Neurotech^EU^ perspective

Daniela Schulz^1†*^, Carmen Lillo-Navarro^2†^, Marc Slors^3^, Anett Hrabéczy^4^, Martin Reuter^5†^

***Correspondence:** Daniela Schulz: daniela.schulz@boun.edu.tr

# Supplementary Table 1: Understanding Societal Characteristics Form (USCF)

**Understanding Societal Characteristics Form (USCF)**

From health and healthcare to learning and education, neuroscience shows great promise to become also an applied science that could benefit society and kindle a new economy in Europe. The European University of Brain and Technology (Neurotech^EU^) aims to be the backbone of this new vision. Neurotech^EU^ was funded by the European Commission to create an ecosystem that fosters neuroscience education, research and innovation, and creates societal impact resulting from the development of new neuro-technologies.

Neurotech^EU^ is a network of 9 universities from across the European Union and Associated Member States: Radboud Universiteit (The Netherlands), Universidad Miguel Hernández de Elche (Spain), Karolinska Institutet (Sweden), Rheinische Friedrich-Wilhelms-Universität Bonn (Germany), Boğaziçi Üniversitesi (Turkey), Universitatea de Medicină și Farmacie „Iuliu Hațieganu” din Cluj-Napoca (Romania), Debreceni Egyetem (Hungary), Université de Lille (France), and Háskólinn í Reykjavík (Iceland), and over 50 associated partners consisting of companies, technology transfer offices, and regional innovation networks, among other.

**As researchers of Neurotech^EU^, our goal is to understand our nations’ perspectives on neuro-technological advances and to integrate these perspectives into the scientific process. Your answers to this form will help us generate statistical data related to the population characteristics of** (***insert your country***). **The data will be compared with those from other Neurotech^EU^ countries. Please answer the questions as viewed by you from your country of residence today.**

_______________________________________________________­­­­­­­­­__________________________

Scroll for page 2

1. **I am __________ years old.**
2. **I am**  □ male □ female □ other
3. **Education (check all that apply)**
   1. **number of years in school (other than university): __________**
   2. **university degrees (check all that apply):** □ Bachelor □ Master □ Ph.D.
4. **Mother’s education**
   1. **number of years in school (other than university): __________**
   2. **university degrees (check all that apply):** □ Bachelor □ Master □ Ph.D.
5. **According to (*insert the office for national statistics of your country*), the mean annual equivalized (for size and composition) household income for (*insert your country*) in 2023 was (*insert net amount for the year and monthly income, in local currency*). My income fell**

□ below □ near □ above this national average

1. **Occupation: ____________________**

□ employed □ retired □ unemployed

1. **Marital status**

□ Single □ Live with partner, not married □ Married □ Married, but separated

□ Divorced □ Widowed

1. **Children**

□ 0 □ 1 □ 2 □ 3 or more

1. **I was born in: ____________________**
2. **I consider myself to be a ____________________ national**
3. **I grew up (check all that apply)**

□ rural □ urban, small city (< 300,000) □ urban, big city (> 300,000)

1. **My current environment is**

□ rural □ urban, small city (< 300,000) □ urban, big city (> 300,000)

1. **Current resident country: ____________________**
2. **I have lived in □ countries for more than 3 months**

□ 1 □ 2 □ 3 □ 4 □ 5 or more

1. **Native language: ____________________**
2. **I speak □ language(s) fluently**

□ 1 □ 2 □ 3 □ 4 □ 5 or more

1. **Do you identify as religious?**

□ Yes □ No

1. **Do you identify as spiritual?**

□ Yes □ No

1. **Do you identify as political?**

□ Yes □ No

1. **My position in politics is**

□ far-left □ left □ center-left □ center □ center-right □ right □ far-right

□ not interested □ don’t want to say

# Supplementary Table 2: Understanding Societal Challenges Questionnaire (USCQ)

**Understanding Societal Challenges Questionnaire (USCQ)**

From health and healthcare to learning and education, neuroscience shows great promise to become also an applied science that could benefit society and kindle a new economy in Europe. The European University of Brain and Technology (Neurotech^EU^) aims to be the backbone of this new vision. Neurotech^EU^ was funded by the European Commission to create an ecosystem that fosters neuroscience education, research and innovation, and creates societal impact resulting from the development of new neuro-technologies.

Neurotech^EU^ is a network of 9 universities from across the European Union and Associated Member States: Radboud Universiteit (The Netherlands), Universidad Miguel Hernández de Elche (Spain), Karolinska Institutet (Sweden), Rheinische Friedrich-Wilhelms-Universität Bonn (Germany), Boğaziçi Üniversitesi (Turkey), Universitatea de Medicină și Farmacie „Iuliu Hațieganu” din Cluj-Napoca (Romania), Debreceni Egyetem (Hungary), Université de Lille (France), and Háskólinn í Reykjavík (Iceland), and over 50 associated partners consisting of companies, technology transfer offices, and regional innovation networks, among other.

**As researchers of Neurotech^EU^, our goal is to understand our nations’ perspectives on neuro-technological advances and to integrate these perspectives into the scientific process. Your answers to this form will help us generate statistical data related to our nations’ needs for, interest in, access to, knowledge of, and trust in neuro-technologies, as well as our views on policymaking regarding their development. The data from your country will be compared with those from other Neurotech^EU^ countries. Please answer the questions as viewed by you from your country of residence today.**

Neuro-technologies encompass a broad range of technologies that allow for a better understanding of how the brain works and help prevent, diagnose, and treat diseases of the brain. Examples include brain scanners and other medical devices, genetic technologies, and artificial intelligence.

_________________________________________________________________________________

Scroll for page 2

**Please read the following statements and indicate to what extent they apply to you:**

1. **Needs for neuro-technological advances**
2. **“Neuro-technologies are important for society.”**

| □ 1 | □ 2 | □ 3 | □ 4 | □ 5 |
| --- | --- | --- | --- | --- |
| strongly disagree | disagree | neither agree nor disagree | agree | strongly agree |

1. **“Society needs new neuro-technologies.”**

| □ 1 | □ 2 | □ 3 | □ 4 | □ 5 |
| --- | --- | --- | --- | --- |
| strongly disagree | disagree | neither agree nor disagree | agree | strongly agree |

- For which purpose(s) or condition(s)? ____________________

1. **“Neuro-technologies play a role in my life.”**

| □ 1 | □ 2 | □ 3 | □ 4 | □ 5 |
| --- | --- | --- | --- | --- |
| strongly disagree | disagree | neither agree nor disagree | agree | strongly agree |

1. **“I have needs for new neuro-technologies.”**

| □ 1 | □ 2 | □ 3 | □ 4 | □ 5 |
| --- | --- | --- | --- | --- |
| strongly disagree | disagree | neither agree nor disagree | agree | strongly agree |

- For which purpose(s) or condition(s)? ____________________

1. **Do you have any neurological condition?**

□ yes □ no

**If yes: “It compromises my daily functioning.”**

| □ 1 | □ 2 | □ 3 | □ 4 | □ 5 |
| --- | --- | --- | --- | --- |
| strongly disagree | disagree | neither agree nor disagree | agree | strongly agree |

1. **Are you undergoing treatment for a neurological condition?**

□ yes □ no

**If yes: “I am complying with the treatment.”**

| □ 1 | □ 2 | □ 3 | □ 4 | □ 5 |
| --- | --- | --- | --- | --- |
| strongly disagree | disagree | neither agree nor disagree | agree | strongly agree |

**Has the level of compliance changed over time?**

□ yes □ no

**If yes, has the level of compliance changed because of (check all that apply)**

□ discomfort with the treatment

□ a change in the severity of the condition

□ other (please state): _______________

1. **Which of these categories apply to you, now or in the past (check all that apply)?**

□ I work in the health care system

□ I have friends/family members who work in the health care system

□ I am a health professional

□ I conduct research in a health-related discipline

□ I am participating in a clinical trial

□ as a patient □ as a caregiver □ as a healthy control □ don’t know

□ I work for a company that develops and/or distributes neuro-technologies

□ None of the above

1. **Interest in neuro-technological advances**
2. **How often do you read about neuro-technologies?**

□ never □ once a year □ once a month □ once a week □ daily

1. **How often do you read about the brain and behavior (any topic)?**

□ never □ once a year □ once a month □ once a week □ daily

1. **How often do you visit science museums or science exhibitions?**

□ never □ once a year □ once a month □ once a week □ daily

1. **How often do you watch web media (e.g. documentaries) about the brain and behavior?**

□ never □ once a year □ once a month □ once a week □ daily

1. **Access to neuro-technological advances**
2. **Where do you most commonly find out about neuro-technologies?**

□ social media □ Wikipedia □ scientific websites □ scientific journals □ TV

□ newspapers □ radio □ ads or posters □ physician’s office □ other □ I don’t care

1. **In how many languages do you follow scientific news?**

□ native language □ in two languages □ in three languages □ in 4+ languages

□ I don’t follow scientific news

1. **Do you use Google translator or other online translators to access articles about science in foreign languages?**

□ yes □ no

1. **“Costs prevent me from accessing neuro-technologies.”**

| □ 1 | □ 2 | □ 3 | □ 4 | □ 5 |
| --- | --- | --- | --- | --- |
| strongly disagree | disagree | neither agree nor disagree | agree | strongly agree |

1. **Which of the following are correct (check all that apply)?**

□ I have to pay for specialized health care, such as neuro-technologies, out of my pocket

□ My health care provider covers specialized health care in part

□ My health care provider covers specialized health care in full

□ I prefer to pay for private health care out of my pocket, but could get public services for free

□ Public health services in my vicinity provide the care I need

1. **“Regulations prevent me from accessing neuro-technologies.”**

| □ 1 | □ 2 | □ 3 | □ 4 | □ 5 |
| --- | --- | --- | --- | --- |
| strongly disagree | disagree | neither agree nor disagree | agree | strongly agree |

1. **Knowledge of neuro-technologies**
2. **Which of the following technologies are you familiar with? Check all that apply.**

□ PC □ tablets □ mobile phones □ wearable technologies □ fitness tracker

□ apple watch □ oximeter □ health apps □ hearing aid □ bionics □ robotics

□ machine learning □ artificial intelligence □ ultrasound □ computed tomography

□ X-ray □ EEG □ magnetic resonance imaging □ positron emission tomography

□ psychotropic drugs □ pharmacotherapy □ implantable drug delivery systems

□ deep brain stimulation □ optogenetics □ CRISPR-Cas9 □ genetics

□ behavior tracking software □ nutrition apps (and/or smart tools, websites, videos)

□ sleep apps □ exercise apps □ exercise equipment □ motion-assistive devices

□ virtual reality

1. **Which of these technologies are currently important for your life or have been in the past? Check all that apply.**

□ PC □ tablets □ mobile phones □ wearable technologies □ fitness tracker

□ apple watch □ oximeter □ health apps □ hearing aid □ bionics □ robotics

□ machine learning □ artificial intelligence □ ultrasound □ computed tomography

□ X-ray □ EEG □ magnetic resonance imaging □ positron emission tomography

□ psychotropic drugs □ pharmacotherapy □ implantable drug delivery systems

□ deep brain stimulation □ optogenetics □ CRISPR-Cas9 □ genetics

□ behavior tracking software □ nutrition apps (and/or smart tools, websites, videos)

□ sleep apps □ exercise apps □ exercise equipment □ motion-assistive devices

□ virtual reality

□ other (please state): _______________

1. **What do you feel about ‘neuro-technologies’? And to what extent? Please circle.**

**1 = low 3 = intermediate 5 = high**

Fear: 1 2 3 4 5

Uncertainty: 1 2 3 4 5

Control by external powers: 1 2 3 4 5

It’s important for medicine: 1 2 3 4 5

It will facilitate our lives: 1 2 3 4 5

Joy: 1 2 3 4 5

Curiosity: 1 2 3 4 5

Relief: 1 2 3 4 5

Helplessness: 1 2 3 4 5

Anger: 1 2 3 4 5

Sadness: 1 2 3 4 5

□ I don’t know

□ Other (please state): _____________

1. **Trust in neuro-technological advances**
2. **“I generally trust the news about neuro-technologies.”**

| □ 1 | □ 2 | □ 3 | □ 4 | □ 5 |
| --- | --- | --- | --- | --- |
| strongly disagree | disagree | neither agree nor disagree | agree | strongly agree |

1. **“I generally trust neuro-technologies that comply with European regulations.”**

| □ 1 | □ 2 | □ 3 | □ 4 | □ 5 |
| --- | --- | --- | --- | --- |
| strongly disagree | disagree | neither agree nor disagree | agree | strongly agree |

1. **“I generally trust U.S. Food and Drug Administration (FDA)-approved neuro-technologies.”**

| □ 1 | □ 2 | □ 3 | □ 4 | □ 5 |
| --- | --- | --- | --- | --- |
| strongly disagree | disagree | neither agree nor disagree | agree | strongly agree |

1. **“I believe that data obtained by neuro-technologies can reveal thoughts or feelings that I would rather keep to myself.”**

| □ 1 | □ 2 | □ 3 | □ 4 | □ 5 |
| --- | --- | --- | --- | --- |
| strongly disagree | disagree | neither agree nor disagree | agree | strongly agree |

1. **“I believe that using neuro-technologies can be a threat to my privacy.”**

| □ 1 | □ 2 | □ 3 | □ 4 | □ 5 |
| --- | --- | --- | --- | --- |
| strongly disagree | disagree | neither agree nor disagree | agree | strongly agree |

1. **“I think neuro-technologies might help me understand myself better.”**

| □ 1 | □ 2 | □ 3 | □ 4 | □ 5 |
| --- | --- | --- | --- | --- |
| strongly disagree | disagree | neither agree nor disagree | agree | strongly agree |

1. **“I believe that data obtained by neuro-technologies can be used or abused for commercial purposes.”**

| □ 1 | □ 2 | □ 3 | □ 4 | □ 5 |
| --- | --- | --- | --- | --- |
| strongly disagree | disagree | neither agree nor disagree | agree | strongly agree |

1. **Perspectives on policymaking**
2. **“Policymakers should follow the recommendations of scientists.”**

| □ 1 | □ 2 | □ 3 | □ 4 | □ 5 |
| --- | --- | --- | --- | --- |
| strongly disagree | disagree | neither agree nor disagree | agree | strongly agree |

1. **“Policymakers should push for the development of new neuro-technologies.”**

| □ 1 | □ 2 | □ 3 | □ 4 | □ 5 |
| --- | --- | --- | --- | --- |
| strongly disagree | disagree | neither agree nor disagree | agree | strongly agree |

1. **“Policymakers should monitor industry as it develops new neuro-technologies.”**

| □ 1 | □ 2 | □ 3 | □ 4 | □ 5 |
| --- | --- | --- | --- | --- |
| strongly disagree | disagree | neither agree nor disagree | agree | strongly agree |
